# Extended investigation of tube-gel sample preparation: a versatile and simple choice for high throughput quantitative proteomics

**DOI:** 10.1038/s41598-018-26600-4

**Published:** 2018-05-29

**Authors:** Leslie Muller, Luc Fornecker, Marie Chion, Alain Van Dorsselaer, Sarah Cianférani, Thierry Rabilloud, Christine Carapito

**Affiliations:** 1 Laboratoire de Spectrométrie de Masse Bio-Organique (LSMBO), IPHC, UMR 7178, Université de Strasbourg, CNRS, 25 rue Becquerel, 67087 Strasbourg, France; 2 Laboratoire de Chimie et Biologie des Métaux, UMR CNRS-CEA-UGA 5249, iRTSV/LCBM, CEA Grenoble, Grenoble, France

## Abstract

Sample preparation for quantitative proteomics is a crucial step to ensure the repeatability and the accuracy of the results. However, there is no universal method compatible with the wide variety of protein extraction buffers currently used. We have recently demonstrated the compatibility of tube-gel with SDS-based buffers and its efficiency for label-free quantitative proteomics by comparing it to stacking gel and liquid digestion. Here, we investigated the compatibility of tube-gel with alternatives to SDS-based buffers allowing notably the extraction of proteins in various pH conditions. We also explored the use of photopolymerization to extend the number of possibilities, as it is compatible with a wide range of pH and is non-oxidative. To achieve this goal, we compared six extraction buffers in combination with two polymerization conditions to further optimize the tube-gel protocol and evaluate its versatility. Identification and quantitative results demonstrated the compatibility of tube-gel with all tested conditions by overall raising quite comparable results. In conclusion, tube-gel is a versatile and simple sample preparation method for large-scale quantitative proteomics applications. Complete datasets are available via ProteomeXchange with identifier PXD008656.

## Introduction

Mass spectrometry (MS) has become a powerful tool for proteomics analysis. Thanks to major advances in recent years in instrumentation and bioinformatics tools, MS-based proteomics analysis allows now identifying and quantifying thousands of proteins from complex proteomes^1^. Bottom-up strategies are the most widely used approaches and rely on the analysis of peptides obtained from digested proteins. Sample preparation and protein digestion are critical steps for quantitative proteomics analysis to ensure the repeatability and robustness of the results. The classical procedure for sample preparation consists in extracting and solubilizing proteins with the use of detergents followed by in-gel or in-solution protein digestion and all these steps are closely interlinked. Sodium dodecyl sulfate (SDS) is among the most efficient and widely used detergents for extraction and solubilisation of proteins, in particular for membrane proteins^2^. However, SDS needs to be removed prior to protein digestion as it would inhibit proteolytic activity of enzymes used for digestion. Moreover, SDS interferes with subsequent liquid chromatography^3^ and MS analysis by inducing ionisation suppression^4,5^. In this context, in-gel approaches are advantageous as the high concentration of SDS employed can be removed by multiple and intensive washing steps^6^. Among in-gel approaches, an original tube-gel (TG) protocol was initially published in 2005 by Lu, X. *et al*.^7^, consisting in directly polymerizing the protein extract in solution and being particularly adapted for the study of membrane proteins^8–12^. In 2016, we have extended the application of TG by demonstrating its compatibility and repeatability for large-scale quantitative proteomics experiments^13^. TG is compatible with high SDS concentrations such as stacking gels, while offering significant gain of time and reduced sample handling with equivalent performances.

If the use of SDS with in-gel digestion could be considered as a classical protocol for sample preparation, many other parameters could be modified, in particular for the removal of DNA or anionic macromolecules. Removal of DNA could be obtained by precipitation with SDS at low pH or with the use of spermine^14,15^, and removal of anionic macromolecules could be obtained with the use of cationic detergents^16,17^. Nevertheless, these conditions are generally not compatible with classical 1D-SDS-PAGE sample preparation methods.

In this context, the goal of our study is to improve our previously published TG protocol and extend its use by exploring its compatibility with various detergents and pH conditions.

## Results and Discussion

Table 1 summarizes the protocols evaluated in the present work and their expected characteristics. The combination of Laemmli buffer (SDS in basic conditions) and chemical polymerization (STC) was considered as the reference protocol against which all other protocols were compared^13^. We investigated alternative extraction buffers based on the use of SDS in acidic condition and cationic detergents such as cetyltrimethylammonium chloride (CTAC) and chaotropic agents (Urea). We also investigated proteins extraction in native conditions with weak denaturation. Acrylamide photopolymerization (PTP) was tested in addition to classical chemical polymerization, to take advantage of its compatibility with a wide range of pH and the absence of oxidizing conditions. All extraction conditions were tested with chemical polymerization and PTP, except for cationic detergents that are not compatible with chemical polymerization because they induce precipitation of ammonium persulfate (APS)^18^. Each condition was prepared in four replicates. Comparisons were done on qualitative data (peptides and proteins identifications) and relative quantitative spectral counting results.

**Table 1:** Summary and expected behavior of the ten tested protocols according to extraction buffer and polymerization type (chemical or photopolymerization) used.

Extraction Buffer	Extraction temperature	Expected behaviour	Protocol name		Conditions
			Chemical Polymerization *(Temed* + *Ammonium Persulfate)*	Photopolymerization *(Methylene blue)*	
SDS/Tris (pH 7.5)	70 °C	Reference protocol [Muller *et al*., 2016]	STC	STP	Basic SDS
SDS/Glycine (pH 2.5)	70 °C	Remove DNA (at pH 2.5 DNA is less negatively charged and precipitate in contact with SDS)	SGC	SGP	Acidic SDS
Urea/Spermine/CHAPS (pH 8)	RT	Remove DNA (Precipitate in contact with Spermine)	USC	USP	Chaotropic agent
CTAC/Glycine (pH 2.5)	70 °C	Remove interfering anionic macromolecules by precipitation	/	CTP	Cationic detergent
CTAC/Glycine/Urea 4 M (pH 2.5)	RT	Remove interfering anionic macromolecules by precipitation	/	CUP	
HEPES/KCl/EDTA/Spermidine/SB3–14	0 °C	Weak denaturation + Remove nucleus/DNA	NAC	NAP	Native

### Qualitative protocols comparison using proteins and peptides identification results

The total number of protein sets identified with at least one unique peptide, after merging results of the four replicates for each protocol, are presented in Table 2. Overall, merged protein numbers ranged from 1838 to 2476, illustrating the suited technical compatibility of TG protocol with all tested conditions. Although the highest number of identified proteins was obtained with the use of STC, large and comparable numbers were also obtained with all other conditions. As expected, the lowest number of identified protein sets was obtained with the native extraction protocol with chemical polymerization. Though, it is noteworthy that despite the weak protein denaturation, the number of identifications remains quite high in native conditions (around 2000 protein sets for the NAP protocol).

**Table 2:** Total number of peptides and protein sets identified with at least one unique peptide in the merged results of the four replicates for each tested protocol.

	STC	STP	SGC	SGP	CTP	CUP	USC	USP	NAC	NAP
Number of identified protein sets with at least one unique peptide	2476	2181	2244	2167	2052	2232	2107	1962	1838	2017
Number of identified peptides unique to a protein set	16092	12425	13018	12905	11954	13308	11189	10937	11205	13583

**Figure 1:**
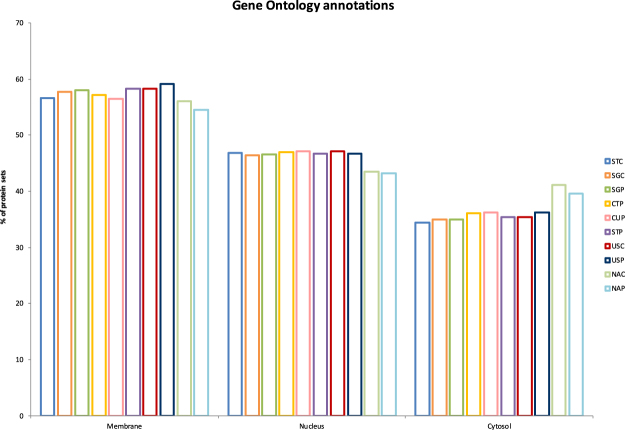
Distributions of the three main Gene Ontology annotations (membrane, nucleus and cytosol, extracted using GO terms listed in Supplementary Table 1) for each protocol obtained after merging the results of the four replicates.

Then, to investigate the impact of the extraction buffer content on the nature of the extracted proteins, we first extracted GO annotations for each protocol after merging identification results of the four replicates (Fig. 1). The distributions of cellular localizations among all extracted proteins with protocols based on the use of detergents or chaotropic agents are roughly comparable, with membrane-annotated proteins ranging from 56 to 59%, nuclear-annotated proteins from 46 to 47% and cytosolic-annotated proteins from 34 to 36%. As expected, the proportion of nuclear proteins was low with the native protocols (43–44%) and the proportion of cytosolic proteins was slightly higher (40–41%), due to the lack of detergents and the removal of nuclei during sample preparation. Secondly, when looking at the physico-chemical properties of the extracted proteins, we did not observe any clear-cut difference regarding hydrophobicity or molecular weight distributions (Supplementary Fig. S1). We only observed a slight difference in the native protocols for isoelectric point distributions (Supplementary Fig. S1) with less basic proteins (pI > 9), possibly explained by a lower number of histone proteins (around 50%) recovered in native protocols thanks to an efficient nuclei removal.

**Figure 2:**
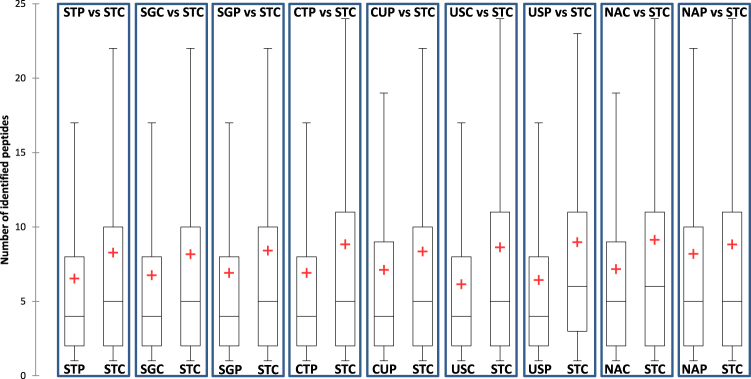
Distributions of the number of peptides for common protein sets between STC and each other protocol.

We compared the number of identified protein sets in STC (considered as the reference) to all other protocols in order to obtain the overlapping proportion of proteins between STC and each other protocols (Table 3). The SDS-based protocols (STP, SGC and SGP) were the closest to STC with more than 75% common protein sets. We also observed a high overlap between STC and other protocols based on the use of urea or cationic detergent (from 68 to 74% common protein sets for CTP, CUP, USC and USP). Also, as expected and due to the difference in the nature of the extracted proteins, the overlap between STC and native protocols was low (60–62%). For each comparison, the quality of the identifications of common proteins was evaluated by plotting the distribution of the number of peptides allowing the identification of these proteins in each protocol (Fig. 2). A higher number of identified peptides was systematically obtained with the STC protocol in each comparison, with median values of 5–6 peptides when considering all the comparisons. The median number of peptides did not differ significantly for all other protocols (with 5 for native conditions and 4 for all the other protocols). These low differences demonstrate the overall equivalent performances of the ten evaluated protocols.

**Table 3:** Number of common and unique proteins identified when comparing STC to each other protocol.

STC versus:	STP	SGC	SGP	CTP	CUP	USC	USP	NAC	NAP
Common proteins (%)	78	79	76	68	74	72	70	60	62
Proteins only identified in STC (%)	17	15	18	24	18	21	25	32	27
Proteins only identified in the other protocol (%)	5	6	6	8	9	7	5	8	11

**Figure 3:**
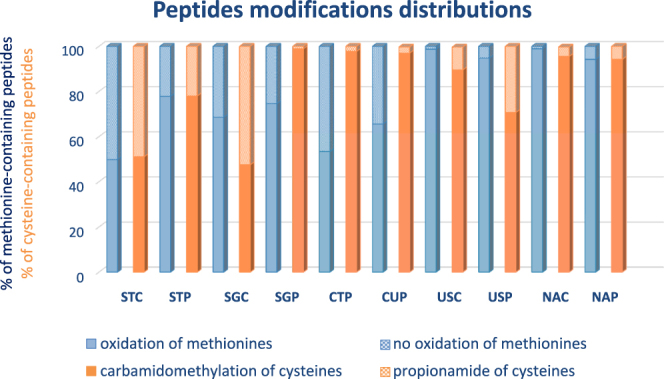
Distributions of peptides modifications occurring on methionine (oxidation) and cysteine residues (carbamidomethylation and propionamide) for each protocol.

At the peptide level, the total number of unique peptides identified in the merged results of the four replicates for each protocol ranged from 10 937 (USP) to 16 092 (STC) (Table 2). Excluding these two extremes, results were equivalent for the other protocols with a mean number of 12 448 identified peptides (with values ranging from 11 189 to 13 583), illustrating that STC slightly outperformed other protocols. We were also interested in studying the modifications of unique peptides induced by each protocol, namely oxidation of methionines and carbamidomethylation and propionamide of cysteines residues. The distributions of these modifications are shown in Fig. 3. We observed that urea and native extractions produced more oxidations of methionines (≥94% of methionine-containing peptides compared to a median of 67% for all other protocols). We hypothesised that one advantage of photopolymerization could be that it generates less oxidation of methionines, but we did not systematically observe this phenomenon when compared to its chemical counterpart. This suggests that oxidations of methionines are influenced by many experimental parameters and not only by the type of polymerization. The combination of SDS-based extraction and chemical polymerization produced most propionamide modifications on cysteines (>50% of cysteine-containing peptides), but it was reduced with PTP (23% for STP and 1% for SGP). We did not observe this phenomenon with urea extraction in which more propionamides were induced by PTP (31%) compared to chemical polymerization (10%). Consequently, the proportion of propionamides differs depending on the extraction buffer and polymerization type used and should definitively be taken into account in all cases for the identification and quantification settings.

### Comparison of the protocols for proteins quantification

In addition to identification results, we extracted quantification results to perform a statistical analysis on spectral count values, again considering STC as the reference protocol (Table 4). We observed few significantly differentially quantified proteins in the SDS-based protocols (≤3% of proteins). Slightly more differentially quantified proteins were found when comparing STC to urea-based protocols (6% for CUP vs STC, 9% for USC vs STC and 8% for USP vs STC). The higher proportion of differentially quantified proteins was obtained when comparing native protocols and CTP to STC (17–19% of proteins). We did not observe any influence depending on the polymerization method, but we can nevertheless conclude that CTP and native protocols are the most different protocols compared to STC.

**Table 4:** Percentage of proteins identified as differentially quantified (p-value < 0.05 and FC >2 or <0.5) by a statistical beta-binomial analysis performed on normalized weighted spectral count values for each comparison of STC versus another protocol.

Comparison of the Beta-Binomial Test	% of Proteins with p < 0.05 and FC > 2	% of Proteins with p < 0.05 and FC < 0.5	% of proteins differentially quantified with p < 0.05 and FC > 2 or < 0.5
STP vs STC	1	1	2
SGC vs STC	1	1	2
SGP vs STC	1	2	3
CTP vs STC	7	10	17
CUP vs STC	2	4	6
USC vs STC	4	5	9
USP vs STC	3	5	8
NAC vs STC	9	10	19
NAP vs STC	8	10	18

Only proteins with at least 5 spectra over the 4 replicates were used.

In order to evaluate more generally the similarity of the different preparation protocols, we have performed a Principal Component Analysis (PCA) and a Hierarchical Clustering on Principle Components (HCPC) (Supplementary Fig. S2). The PCA illustrates a perfect clustering of the different technical replicates, while native protocols clearly discriminate in dimension 1 and CTAC-based protocols in dimension 2. When checking major contributors at the dimension 1, histone H4 protein (P62806) reveals to be the top hit, which is in line with the efficient nuclei removal in the native protocols. Similarly, the HCPC classification shows a clear clustering of the technical replicates and a major discrimination of the native protocols.

### Evaluation of the repeatability of the protocols

The technical repeatability of each protocol was first evaluated by considering the number of protein sets identified (with at least one unique peptide) across the four replicates (Supplementary Fig. S3) and the proportions of common proteins, ranging from 56 to 61% when considering proteins common in the four replicates, and from 69 to 72% when considering proteins in common in at least 3 out of the 4 replicates (Supplementary Fig. S4). We also evaluated the repeatability at the peptide level by considering the number of peptides (unique to a protein set) identified across the four replicates, and the proportions of common peptides between the four replicates per protocols illustrated by venn diagramms (Supplementary Figs S5 et S6). Proportions of common peptides ranged from 42% to 46% between the four replicates and from 58 to 61% in at least 3 out of the 4 replicates. These results illustrated a satisfying technical repeatability at both proteins and peptides levels for all evaluated extraction/polymerization protocols. This is even emphasized by the low numbers of singleton (on average 5 and 6% for proteins and peptides respectively) (Supplementary Figs S4 and S6). Then, we evaluated the impact of the sample preparation variability on the GO annotations distributions for the four replicates (Supplementary Fig. S7) and results are overall consistent attesting again for a good technical repeatability.

**Figure 4:**
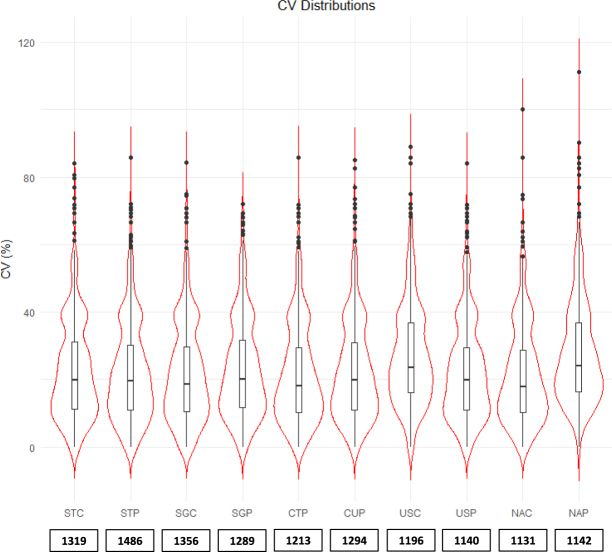
Violin plots indicating the distributions of CV for each protocol calculated for the four replicates using weighted spectral count values. Only proteins with at least one specific spectral count in each replicate were taken into account. The number of proteins included in the calculation of the CV are indicated under each violin plot.

Finally, we evaluated the repeatability at the quantitative level, by plotting the CV distributions for each protocol calculated over the four replicates using weighted spectral count values (Fig. 4). For most protocols, the median CV value was around 20%, with highest values obtained with USC and NAP protocols (24% in both cases), indicating a good repeatability of all protocols.

### Concluding remarks

Extraction and solubilization of proteins in proteomics workflow relies mostly on the use of urea or SDS because of the lack of established sample preparation protocols compatible with other detergents. However, other detergents could be of interest, like CTAC, which allows removal of interfering macromolecules, and also native extractions if samples have to be compatible for further biochemical analyses. We previously demonstrated that TG, in combination with the use of SDS, is a fast and effective sample preparation method for high-throughput quantitative proteomics. Here we showed that TG is a versatile sample preparation because of its wide compatibility with urea, SDS, CTAC and native-based extraction buffers. The reference protocol, namely using SDS and chemical polymerization, allowed identifying the highest number of proteins and peptides, but all the other tested protocols compared favourably in terms of identification, quantification and repeatability. Even if major proportions of proteins are conserved, each extraction/polymerization protocol has its specificities with the native protocols allowing to target a slightly different population of proteins thanks to nuclei removal. CTAC-based protocols, and in particular CTP, also slightly discriminate very likely due to an efficient removal of interfering anionic macromolecules. In conclusion, TG is a fast, simple and reproducible sample preparation compatible with a wide range of extraction buffers offering flexibility during the proteomics sample preparation step. And, as all the extraction protocols compared in this study involved the same number of steps and solutions, it can be reasonably inferred that the observed differences stem mostly from the different chemistries used.

### Recommendations for use

It must be kept in mind that during the tube gel preparation protocol, all compounds initially present in the sample remain in the final gel plug, so that undesirable effects may occur arising from the sample composition. In particular, thiol-containing compounds strongly inhibit gel polymerization, and thiourea inhibits persulfate-initiated polymerization^19^ (but not photopolymerization).

Besides this basic effect, it must be kept in mind that non-proteinaceous, acidic molecules (e.g. nucleic acids, anionic polysaccharides) are likely to inhibit protein digestion (by binding trypsin) and/or peptide extraction by electrostatic effects. The overall efficiency of the process may thus greatly vary according to the sample composition, and the ability of cationic detergents to precipitate such anionic macromolecules^16^ may be advantageous for samples rich in anionic macromolecules.

## Materials and Methods

### Cell culture and protein extraction

J774 cells (obtained from ECACC, Salisbury, UK) were grown in RPMI1640 medium supplemented with 10% fetal bovine serum up to a cell density of 1 million cells/ml. The cells coming from several culture flasks were harvested by scraping and washed 3 times in PBS. The pellets were then resuspended in PBS, the cell suspensions pooled and then split into 6 aliquots for the various cell extractions. All extractions took place with five volumes of extraction buffer per volume of packed cell pellet. This scheme ensures that the comparison of the proteomic results is not biased by a biological variation in the starting material.

(i) Native extraction (NA)
  The cell pellet was resuspended by vortexing in the native lysis buffer (Hepes-NaOH 10 mM pH 7.5, EDTA 1 mM, Spermidine trihydrochloride 1 mM, KCl 50 mM, tetradecyldimethylammonio propane sulfonate (SB 3–14) 0.1% w/v) and then incubated on ice for 20 min with occasional vortexing. The suspension was then centrifuged for 5 min at 2000 g to pellet the nuclei, and the supernatant was collected. The protein concentration was measured by a dye binding assay^20^.
(ii) Urea extraction (US)
  The cell pellet was resuspended by vortexing in the urea lysis buffer (urea 8 M, CHAPS 3%, spermine base 30 mM, HCl 100 mM), and the extraction was let to proceed at room temperature for 30 min. The nucleic acids were then removed by centrifugation for 15 min at 15,000 g and the supernatant was collected. The protein concentration was measured by a dye binding assay^20^.
(iii) Basic SDS extraction (ST)
  The cell pellet was resuspended by vortexing in the neutral SDS lysis buffer (SDS 2%, Tris HCl 100 mM pH 7.5). The extraction took place in a water bath at 70 °C for 30 min. After cooling, the viscosity of the extract was decreased by passing the viscous extract in a 1 ml syringe fitted with a 23 gauge needle. The protein concentration was measured by a modified, detergent-compatible dye binding assay^21^.
(iv) Acidic SDS extraction (SG)
  The cell pellet was resuspended by vortexing in the acidic SDS lysis buffer (SDS 2%, glycine 100 mM, HCl 50 mM, pH 2.4). The extraction took place in a water bath at 70 °C for 30 min. After cooling, the extract was centrifuged for 15 min at 15,000 g and the supernatant was collected. The protein concentration was measured by a modified dye binding assay^21^.
(v) Acidic CTAC extraction (CT)
  The cell pellet was resuspended by vortexing in the acidic CTAC lysis buffer (hexadecyl trimethylammonium chloride (CTAC) 2.5%, glycine 100 mM, HCl 50 mM, pH 2.4). The extraction took place in a water bath at 70 °C for 30 min. After cooling, the extract was centrifuged for 15 min at 15,000 g and the supernatant was collected. The protein concentration was measured by a modified dye binding assay^21^.
(vi) CTAC-urea extraction (CU)

The cell pellet was resuspended by vortexing in the CTAC-urea lysis buffer (urea 4 M, CTAC 2.5%, glycine 100 mM, HCl 50 mM, pH 2.4). The extraction took place at room temperature for 30 min. The extract was then centrifuged for 15 min at 15,000 g and the supernatant was collected. The protein concentration was measured by a modified dye binding assay^21^.

### Tube gel preparation

For each condition, independent quadruplicate gels plugs were prepared. The gel plugs were cast in 1.5 mL Eppendorf tubes. The protein amount was set at 100 µg, the acrylamide concentration was set at 10% and the gel plug volume was set at 100 µL. All initiator solutions (except the dye solutions) were made fresh the day of use. This scheme has been selected to mimic a real comparative proteomic experiment, in which different samples will be in different gel plugs. For each type of sample, it also investigated the variability brought by the proteomic process on a single starting sample. However, the variability brought by the extraction process itself, within each extraction type, was not investigated by this scheme.

### Chemical polymerization

The polymerization was initiated by adding 4 µL of diluted TEMED (dilution: 40 fold in water) and 4 µL of 2.5% ammonium persulfate to the sample/acrylamide/water mixture. The polymerization was left to proceed for 1.5 hour at room temperature.

Native, urea and neutral SDS samples can be polymerized in this format without any further adjustment. Acidic SDS samples were first brought back to neutrality by adding an identical volume of Tris 100 mM to the sample volume. CTAC-containing samples cannot be polymerized by this method irrespective of the pH because of the CTAC-persulfate precipitation^18^, which leads to polymerization inhibition^22^.

### Photopolymerization

The polymerization was initiated by adding 5 µL of 25 mM sodium toluene sulfinate, 5 µL of 0.5 mM diphenyliodonium chloride and 5 µL of 0.5 mM dye solution. Photopolymerization was carried out for 2 hours with a 20 W LED lamp placed 30 cm above the tubes. Initial tests compared methylene blue^23^, eosin Y and flavine mononucleotide^24^ as initiator dyes. Eosin was selected for its ability to perform well with all types of extracts.

### Gel plugs post-treatment

After polymerization, the gel plugs were fixed for 2 × 1 hour in 2% phosphoric acid, 30% ethanol, and then shrunk in 30% ethanol for 1 hour, prior to storage at −20 °C in 30% ethanol. To ensure proper fixation, the fixative was added on top of the gel plug, gel plug was lifted with a clean gel loading plastic tip and the tube was placed upside down on a rocking table (1 stroke per second).

### In-gel digestion

The gel plugs were cut in 2 mm sections and each section in ~2 mm² pieces, prior to in-gel digestion in a 2 mL Eppendorf tube. The gel pieces were washed four times with 400 μL of 75% ACN and 25% NH_4_HCO_3_ at 25 mM and dehydrated with 400 μL of ACN. The cysteine residues were reduced by adding 10 mM DTT for 30 min at 60 °C and 30 min at room temperature, and alkylated by adding 55 mM IAA for 20 min in the dark. The bands were then washed three times by adding 200 μL of 25 mM NH_4_HCO_3_ and 200 μL of ACN. After two dehydrations with 200 μL of ACN, the proteins were cleaved in an adequate volume to cover all gel pieces with a modified porcine trypsin (Promega) solution at a 1:100 w/w enzyme:protein ratio. Digestion was performed overnight at 37 °C. Tryptic peptides were extracted twice under agitation, first with 160 μL of 60% ACN in 0.1% FA for 1 h and then with 160 µL of 100% ACN for 30 min. The collected extracts were pooled, the excess ACN was vacuum dried, and the samples were resolubilized with 150 µL of H2O/ACN/FA (98/2/0.1 v/v/v).

### NanoLC-MS/MS Analysis

NanoLC-MS/MS analysis was performed on a nanoAcquity UPLC device (Waters, Milford, USA) coupled to a TripleTOF 5600 mass spectrometer (Sciex, Framingham, MA, USA). Peptide separation was performed on an ACQUITY UPLC BEH130 C18 column (250 mm × 75 μm with 1.7 μm diameter particles) and a Symmetry C18 precolumn (20 mm × 180 μm with 5 μm diameter particles, Waters). The solvent system consisted of 0.1% FA in water (solvent A) and 0.1% FA in ACN (solvent B). The samples (1 μL) were loaded into the enrichment column over 3 min at 5 μL/min with 99% of solvent A and 1% of solvent B. The peptides were eluted at 300 μL/min with the following gradient of solvent B: from 3 to 35% over 110 min, and 35 to 85% over 5 min.

The Ion Spray Voltage Floating was set to 2.6 kV and the interface heater at 100 °C. The system was operated in data-dependent-acquisition mode with automatic switching between MS (mass range 400–1250 m/z) and MS/MS (mass range 100–1800 m/z in high sensitivity mode) modes. The fifty most abundant peptides (intensity threshold of 150 counts), were selected on each MS spectrum for further isolation and collision induced dissociation fragmentation, preferably from 2+ to 5+ charged ions. The dynamic exclusion time was set to 6 s. Complete datasets are available via ProteomeXchange with identifier PXD008656.

### Data analysis

Raw data were converted into calibrated peaklists.mgf using ProteinPilot™ software (v. 5.0) before being subjected to a search against a concatenated target-decoy database including both forward (target) and reversed (decoy) UniProtKB-SwissProt mouse sequences (17 August 2016, 33580 total entries) using Mascot search algorithm (v.2.5.1). Searches were performed with a mass measurement tolerance of 15 ppm for precursor and 0.05 Da for fragment ions. Oxidation of methionine residues, carbamidomethylation as well as propionamidation of cysteine residues were searched as variable modifications. A maximum of one missed cleavage was allowed. Proline was used to validate the identification results and to perform spectral count quantification. For each sample, Peptide Spectrum Matches were filtered out according to following criteria: pretty rank >1, ion score <25, peptide length <7 residues and a maximum false discovery rate of 1% on the adjusted e-value. Then, proteins were filtered out in order to obtain a final list with a maximum false discovery rate of 1% based on the modified protein MudPit score. The results of the four replicates per protocol were merged in order to compare the protocols. Gene Ontology annotations were extracted from UniProt for each protein. We only consider proteins identified with at least one unique peptide. Quantification by Spectral Count was performed using the list of identified proteins obtained by merging all protocol merges. For the CV calculation, only proteins with at least one specific spectral count in each replicate were taken into account and weighted spectral count values of the four replicates for each protocol were used. For statistical analysis, only proteins identified with at least 5 spectra over the 4 replicates were considered and weighted spectral count values normalized according to the total number of spectra for each sample were used. A beta-binomial test was performed to compare each protocol to STC using an in house-developed software (MSDA: Mass Spectrometry Data Analysis, https://msda.unistra.fr)^25^. For each comparison, proteins were considered as variant if the *p-*value was less than 0.05 and if the calculated fold-change was below 0.5 or higher than 2. The following statistical analysis was done using the 1.1.447 version of R-Studio under the 3.4.4 version of R software. A Principle Component Analysis (PCA) and a Hierarchical Clustering on Principle Components (HCPC) were performed assuming the proteins as variables and the protocols as individuals. The PCA was carried out using the PCA function from the FactoMineR package (1.40 version) with default parameters. The barplots describing the protein contributions to the principle components were obtained with the fviz_contrib function from the factoextra package (1.05 version), setting the choice parameter to ‘var’, the axes parameter to 1 for the first dimension and 2 for the second one. The HCPC was performed using the HCPC function from the FactoMineR package (1.40 version) and defining the nb.clust parameter to −1.

## Electronic supplementary material

Supplementary Figures and Table
