# Supplementary material for: Extended investigation of tube-gel sample preparation: a versatile and simple choice for high throughput quantitative proteomics
Source: Sci Rep. 2018 May 29;8:8260. doi: 10.1038/s41598-018-26600-4 (PMC5974325; doi:10.1038/s41598-018-26600-4)
Supplement: Supplementary file 1 — Supplementary Figures and Table [file 41598_2018_26600_MOESM1_ESM.pdf]

Extended investigation of tube-gel sample preparation: a versatile and simple choice for high throughput quantitative proteomics

Leslie Muller[1+], Luc Fornecker[1+], Marie Chion[1], Alain Van Dorsselaer[1], Sarah Cianférani[1], Thierry Rabilloud[2], Christine Carapito[1*]

[1]Laboratoire de Spectrométrie de Masse Bio-Organique (LSMBO), IPHC, UMR 7178, Université de Strasbourg, CNRS, 25 rue Becquerel, 67087 Strasbourg, France

[2]Laboratoire de Chimie et Biologie des Métaux, UMR CNRS-CEA-UGA 5249, iRTSV/LCBM, CEA Grenoble, Grenoble, France.

Supplementary Figures

Supplementary Figure S1

Distributions of protein molecular weight (**a**) and isoelectric points (**b**) obtained from https://web.expasy.org/compute_pi/, and GRAVY number (**c**) obtained from http://gravy-calculator.de of the proteins identified with at least one unique peptide in the merged results of the four replicates for each protocol.

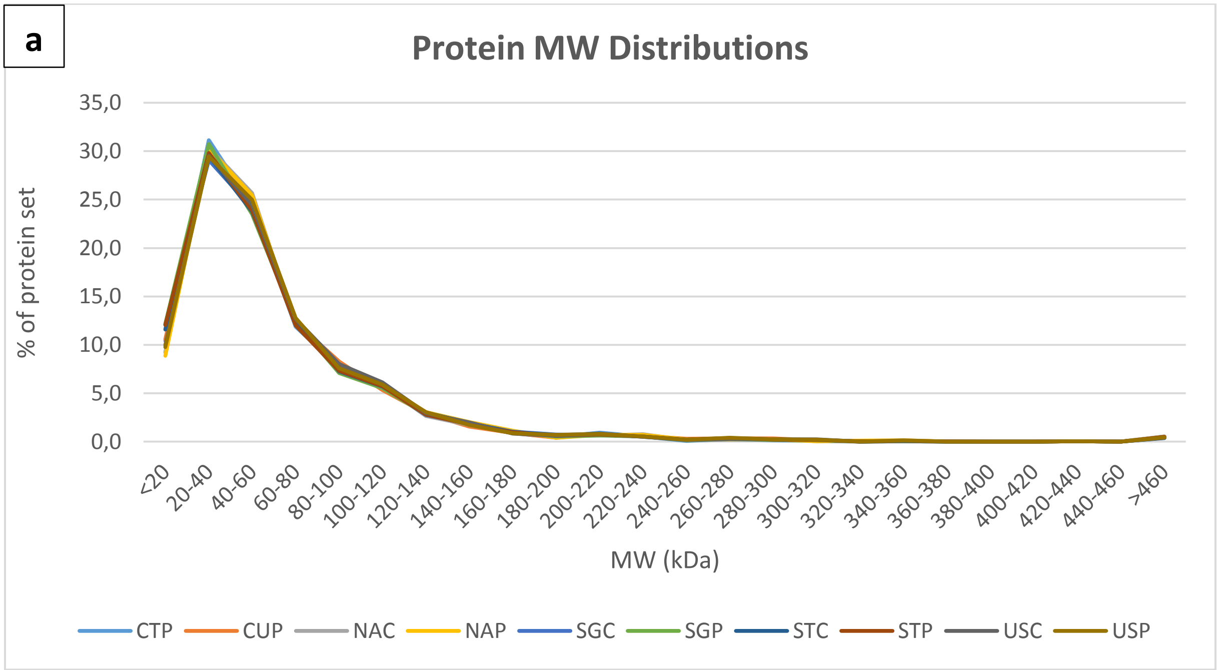

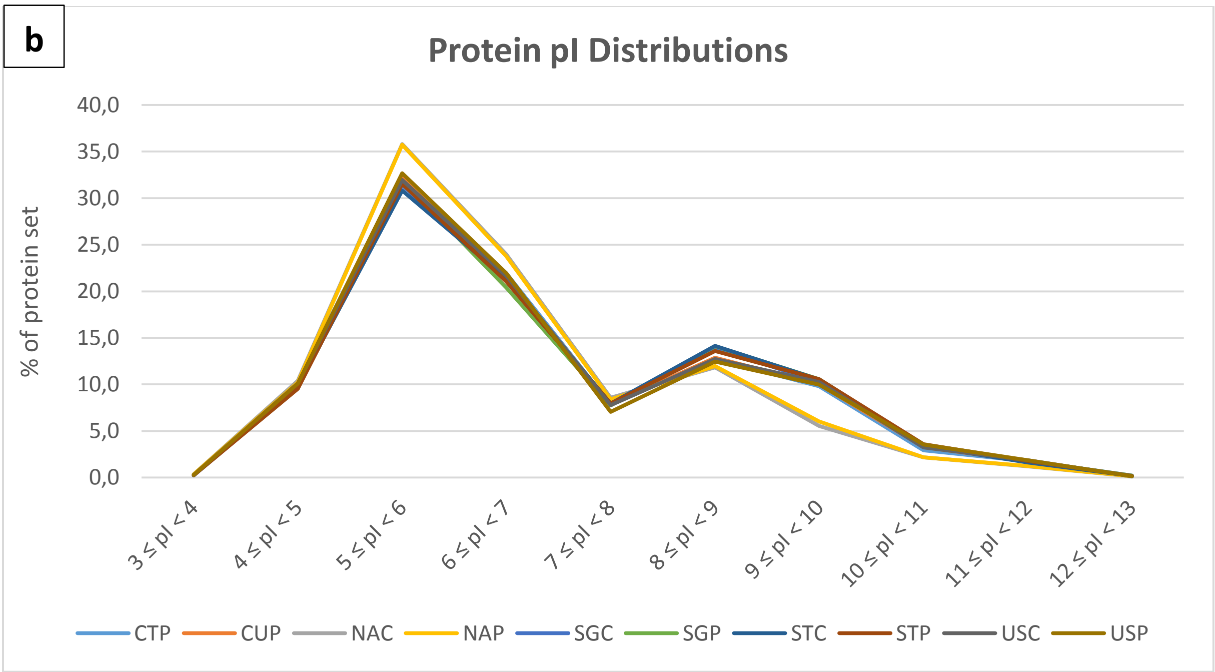

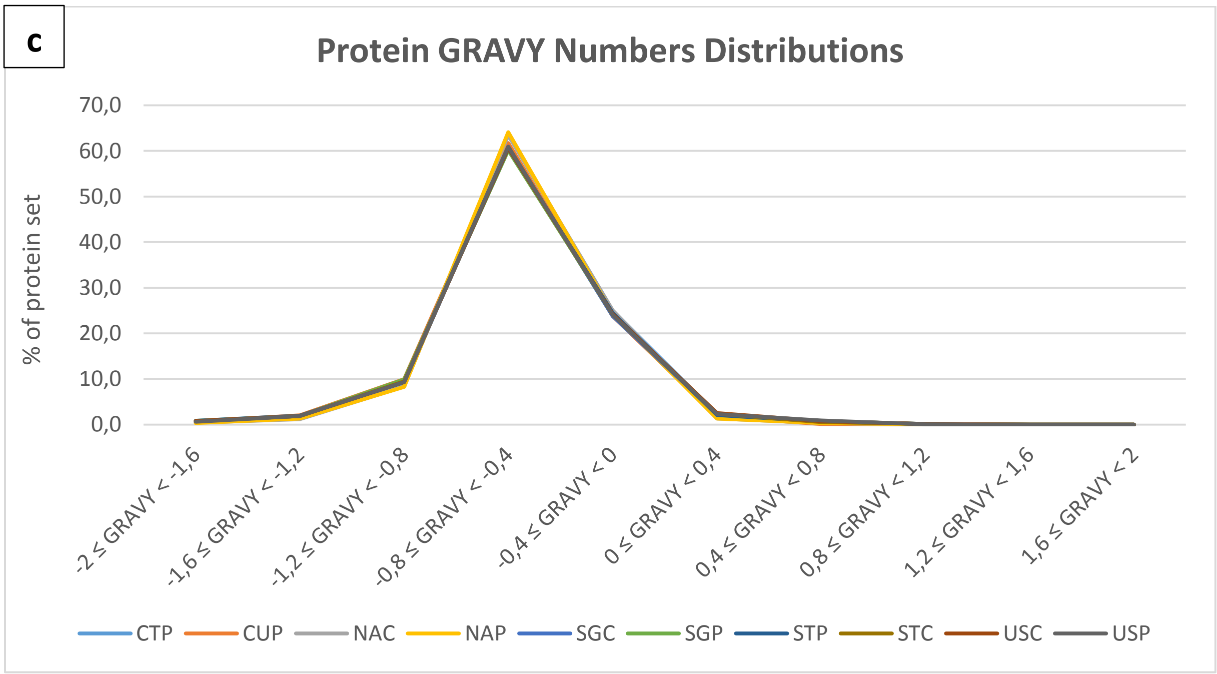
c
Protein GRAVY Numbers Distributions
% of protein set
70,0
60,0
50,0
40,0
30,0
20,0
10,0
0,0
-2 ≤ GRAVY < -1,6
-1,6 ≤ GRAVY < -1,2
-1,2 ≤ GRAVY < -0,8
-0,8 ≤ GRAVY < -0,4
-0,4 ≤ GRAVY < 0
0 ≤ GRAVY < 0,4
0,4 ≤ GRAVY < 0,8
0,8 ≤ GRAVY < 1,2
1,2 ≤ GRAVY < 1,6
1,6 ≤ GRAVY < 2
CTP
CUP
NAC
NAP
SGC
SGP
STP
STC
USC
USP

Supplementary Figure S2

Principle Components Analysis plot (**a**) and Hierarchical Clustering on Principle Components (**b**) performed on normalized weighted spectral count values. Only proteins with at least 5 spectra over the 4 replicates were used.

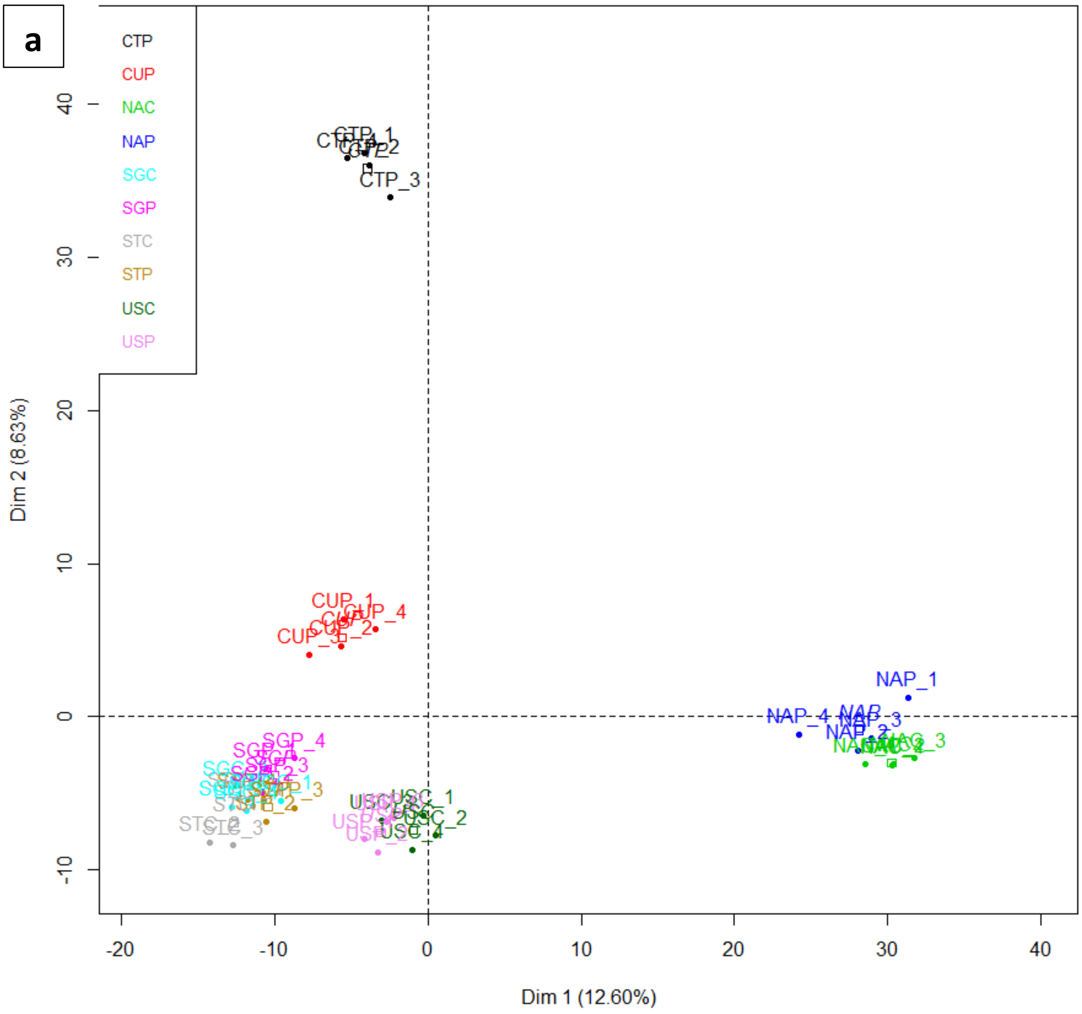

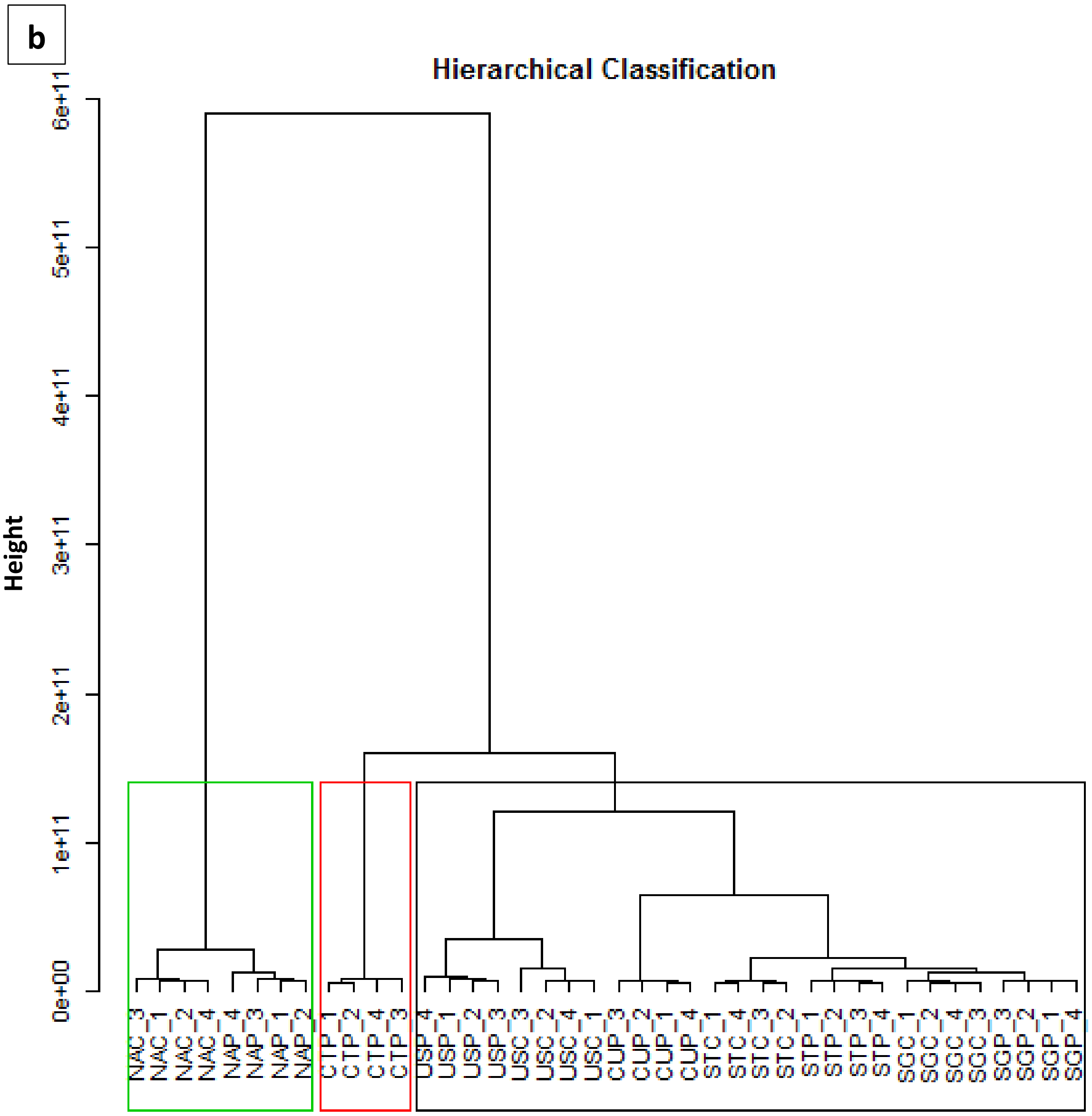
b
Hierarchical Classification
Height
0e+00
1e+11
2e+11
3e+11
4e+11
5e+11
6e+11
NAC_3
NAC_1
NAC_2
NAC_4
NAP_4
NAP_3
NAP_1
NAP_2
CTP_1
CTP_2
CTP_4
CTP_3
USP_4
USP_1
USP_2
USP_3
USC_3
USC_2
USC_4
USC_1
CUP_3
CUP_2
CUP_1
CUP_4
STC_1
STC_4
STC_3
STC_2
STP_1
STP_2
STP_3
STP_4
SGC_1
SGC_2
SGC_4
SGC_3
SGP_3
SGP_2
SGP_1
SGP_4

Supplementary Figure S3

Number of identified protein sets, with at least one unique peptide in each replicate for each protocol.

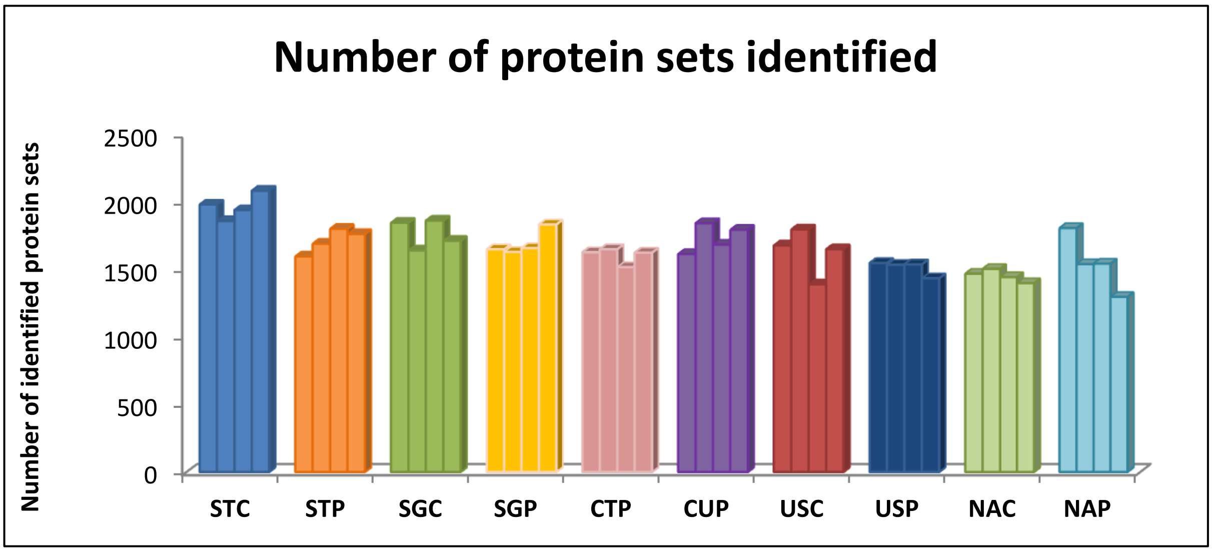

Supplementary Figure S4

Venn Diagrams showing the common proteins between replicates for each evaluated protocol. The red circle indicates the number of proteins identified in 4 out of 4 replicates. The green circles, in addition to the red circle, indicate the number of proteins identified in at least 3 out of the 4 replicates.

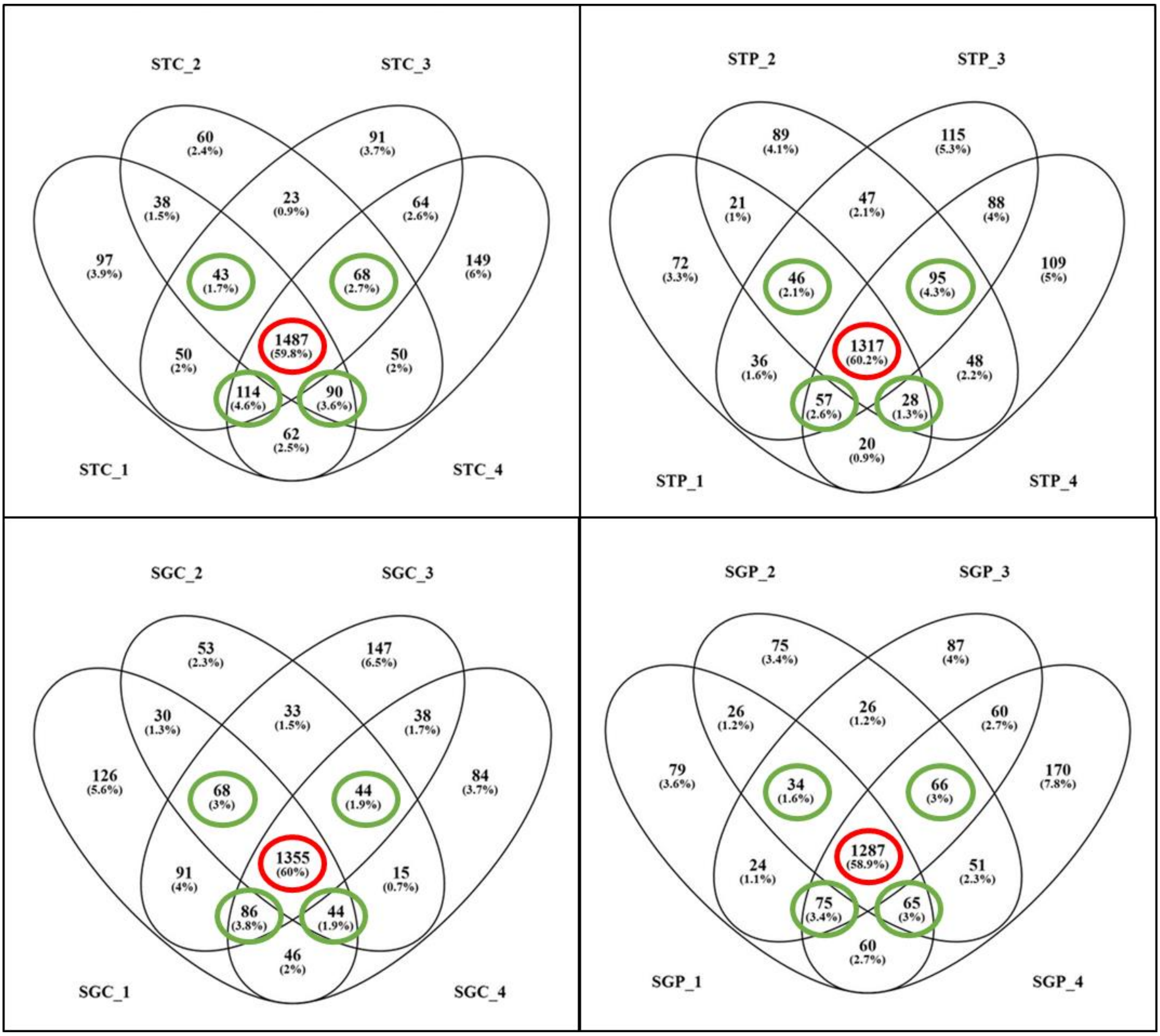

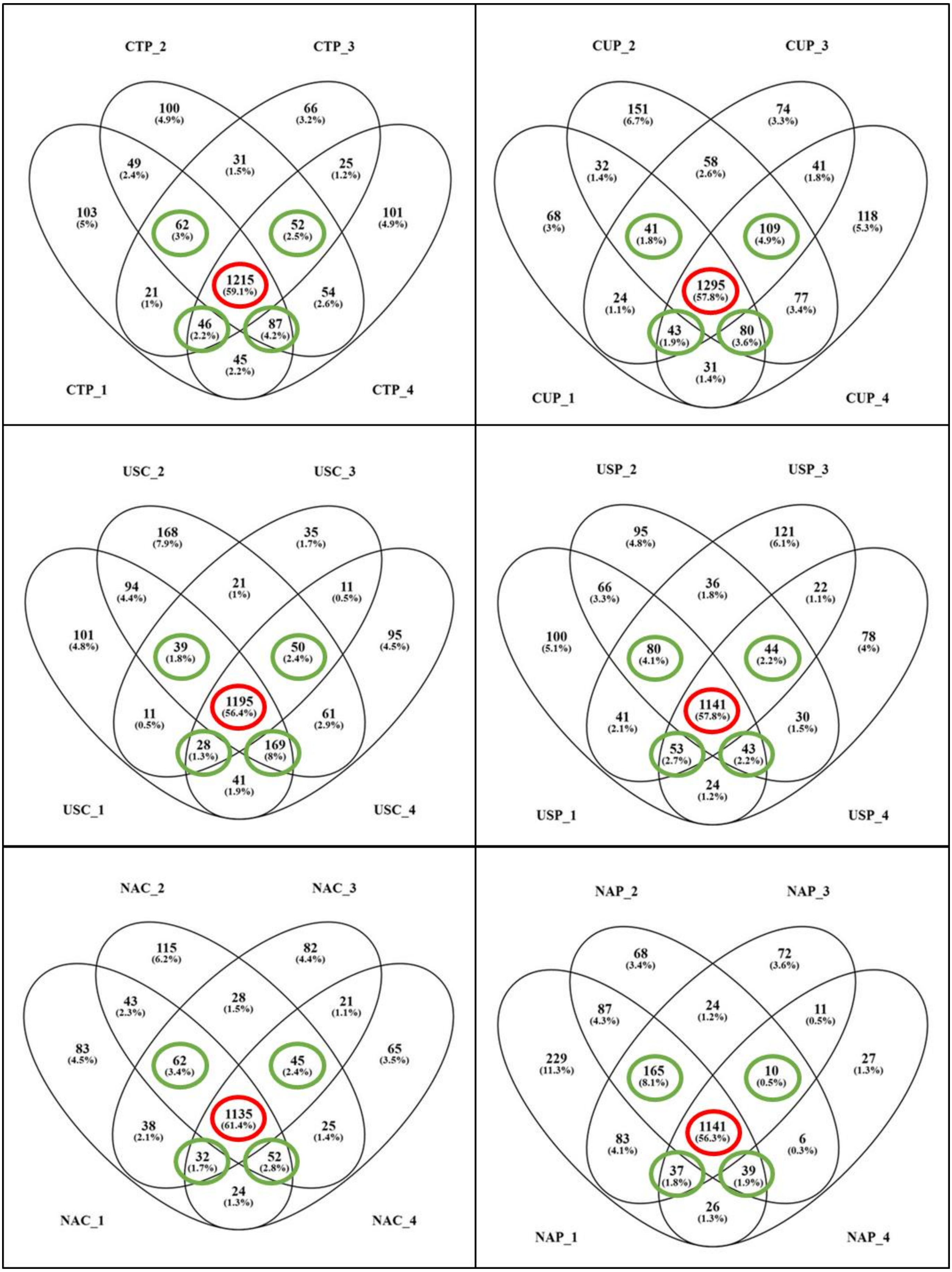
CTP_2
CTP_3
100 (4.9%)
66 (3.2%)
49 (2.4%)
31 (1.5%)
25 (1.2%)
103 (5%)
62 (3%)
52 (2.5%)
101 (4.9%)
1215 (59.1%)
21 (1%)
54 (2.6%)
46 (2.2%)
87 (4.2%)
45 (2.2%)
CTP_1
CTP_4
CUP_2
CUP_3
151 (6.7%)
74 (3.3%)
32 (1.4%)
58 (2.6%)
41 (1.8%)
68 (3%)
41 (1.8%)
109 (4.9%)
118 (5.3%)
1295 (57.8%)
24 (1.1%)
77 (3.4%)
43 (1.9%)
80 (3.6%)
31 (1.4%)
CUP_1
CUP_4
USC_2
USC_3
168 (7.9%)
35 (1.7%)
94 (4.4%)
21 (1%)
11 (0.5%)
101 (4.8%)
39 (1.8%)
50 (2.4%)
95 (4.5%)
1195 (56.4%)
11 (0.5%)
61 (2.9%)
28 (1.3%)
169 (8%)
41 (1.9%)
USC_1
USC_4
USP_2
USP_3
95 (4.8%)
121 (6.1%)
66 (3.3%)
36 (1.8%)
22 (1.1%)
100 (5.1%)
80 (4.1%)
44 (2.2%)
78 (4%)
1141 (57.8%)
41 (2.1%)
30 (1.5%)
53 (2.7%)
43 (2.2%)
24 (1.2%)
USP_1
USP_4
NAC_2
NAC_3
115 (6.2%)
82 (4.4%)
43 (2.3%)
28 (1.5%)
21 (1.1%)
83 (4.5%)
62 (3.4%)
45 (2.4%)
65 (3.5%)
1135 (61.4%)
38 (2.1%)
25 (1.4%)
32 (1.7%)
52 (2.8%)
24 (1.3%)
NAC_1
NAC_4
NAP_2
NAP_3
68 (3.4%)
72 (3.6%)
87 (4.3%)
24 (1.2%)
11 (0.5%)
229 (11.3%)
165 (8.1%)
10 (0.5%)
27 (1.3%)
1141 (56.3%)
83 (4.1%)
6 (0.3%)
37 (1.8%)
39 (1.9%)
26 (1.3%)
NAP_1
NAP_4

Supplementary Figure S5

Number of identified peptides (unique to a protein set) in each replicate for each protocol.

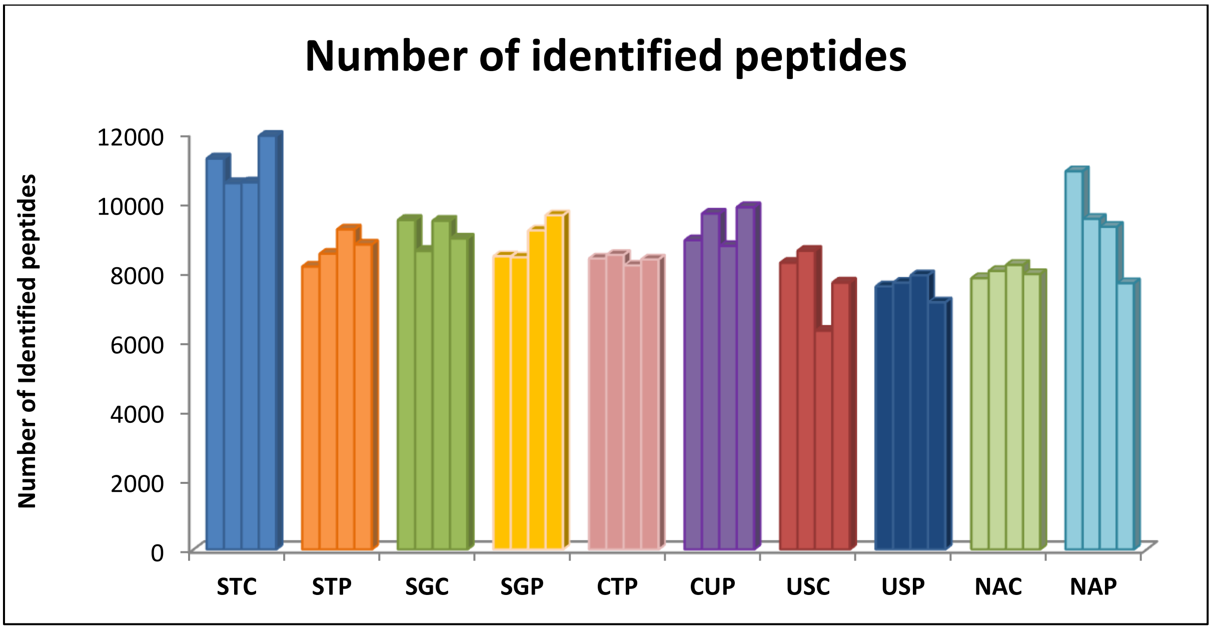

Supplementary Figure S6

Venn Diagrams showing the common peptides between replicates for each protocol method. The red circle indicates the number of peptides identified in 4 out of 4 replicates. The green circles, in addition to the red circle, indicate the number of peptides identified in at least 3 replicates.

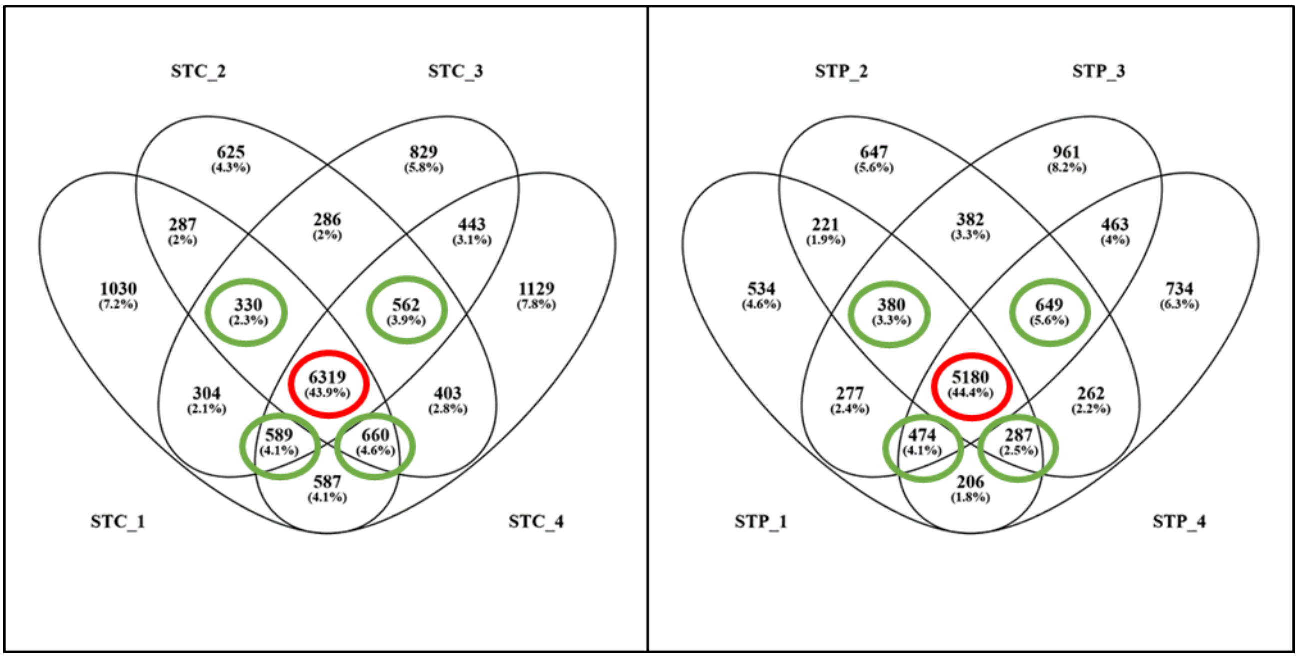

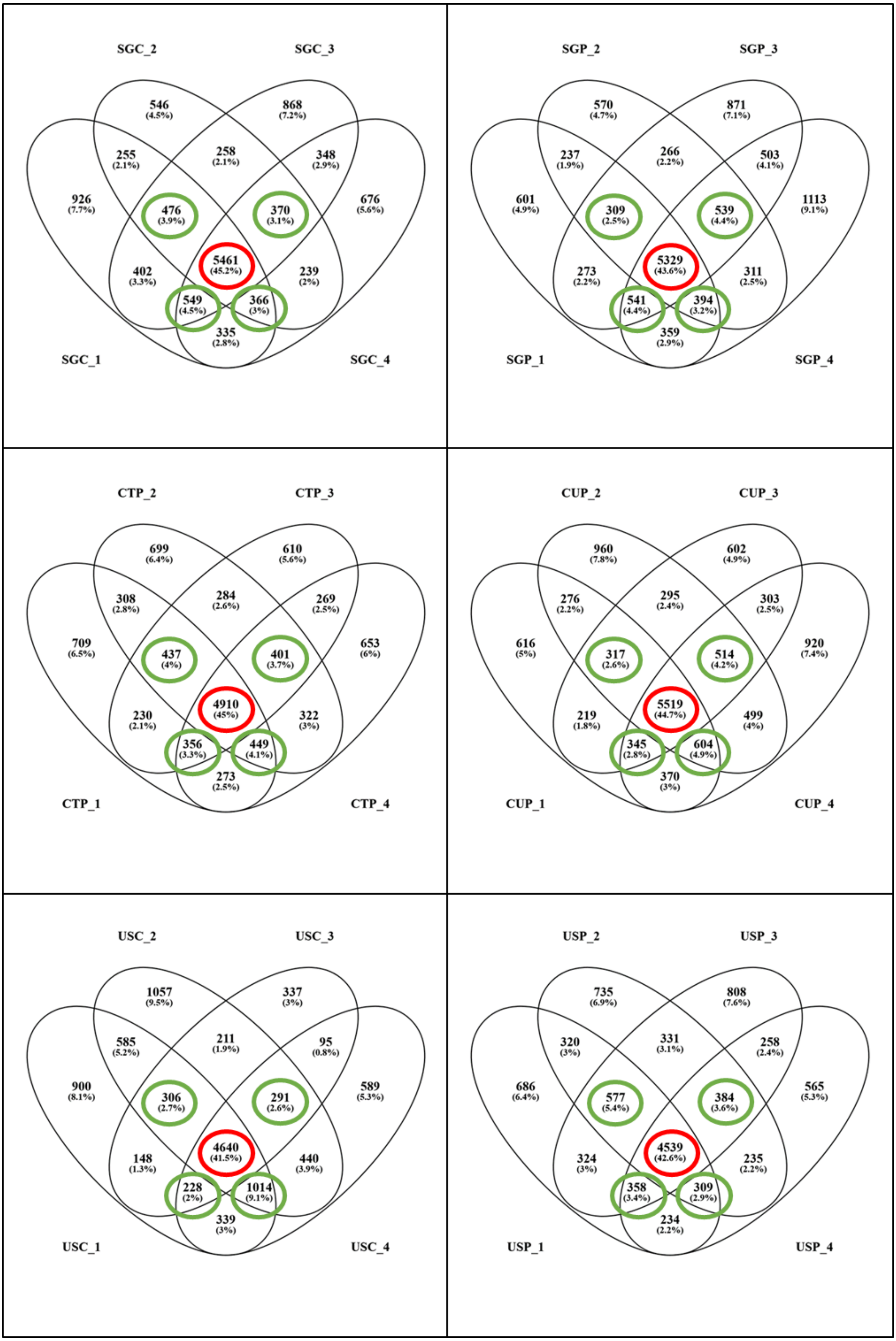
SGC_2
SGC_3
546 (4.5%)
868 (7.2%)
255 (2.1%)
258 (2.1%)
348 (2.9%)
926 (7.7%)
476 (3.9%)
370 (3.1%)
676 (5.6%)
5461 (45.2%)
402 (3.3%)
239 (2%)
549 (4.5%)
366 (3%)
335 (2.8%)
SGC_1
SGC_4
SGP_2
SGP_3
570 (4.7%)
871 (7.1%)
237 (1.9%)
266 (2.2%)
503 (4.1%)
601 (4.9%)
309 (2.5%)
539 (4.4%)
1113 (9.1%)
5329 (43.6%)
273 (2.2%)
311 (2.5%)
541 (4.4%)
394 (3.2%)
359 (2.9%)
SGP_1
SGP_4
CTP_2
CTP_3
699 (6.4%)
610 (5.6%)
308 (2.8%)
284 (2.6%)
269 (2.5%)
709 (6.5%)
437 (4%)
401 (3.7%)
653 (6%)
4910 (45%)
230 (2.1%)
322 (3%)
356 (3.3%)
449 (4.1%)
273 (2.5%)
CTP_1
CTP_4
CUP_2
CUP_3
960 (7.8%)
602 (4.9%)
276 (2.2%)
295 (2.4%)
303 (2.5%)
616 (5%)
317 (2.6%)
514 (4.2%)
920 (7.4%)
5519 (44.7%)
219 (1.8%)
499 (4%)
345 (2.8%)
604 (4.9%)
370 (3%)
CUP_1
CUP_4
USC_2
USC_3
1057 (9.5%)
337 (3%)
585 (5.2%)
211 (1.9%)
95 (0.8%)
900 (8.1%)
306 (2.7%)
291 (2.6%)
589 (5.3%)
4640 (41.5%)
148 (1.3%)
440 (3.9%)
228 (2%)
1014 (9.1%)
339 (3%)
USC_1
USC_4
USP_2
USP_3
735 (6.9%)
808 (7.6%)
320 (3%)
331 (3.1%)
258 (2.4%)
686 (6.4%)
577 (5.4%)
384 (3.6%)
565 (5.3%)
4539 (42.6%)
324 (3%)
235 (2.2%)
358 (3.4%)
309 (2.9%)
234 (2.2%)
USP_1
USP_4

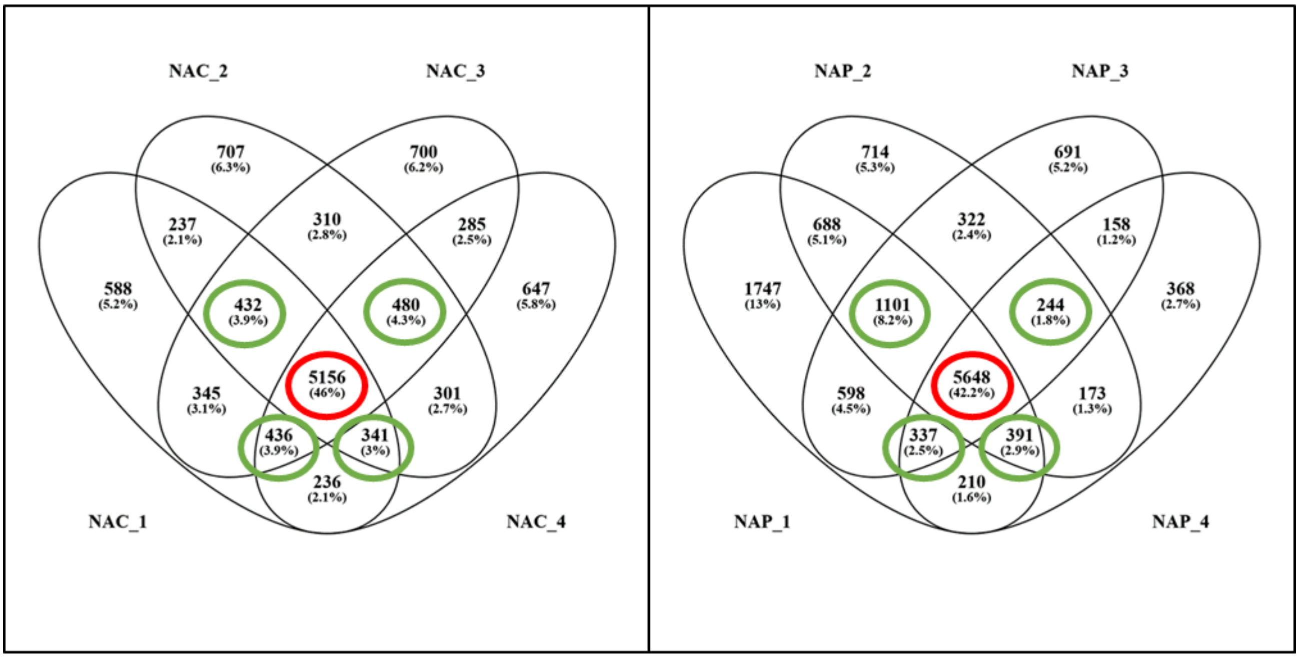

Supplementary Figure S7

Average distributions of the four replicates for each protocol for the three main Gene Ontology annotations (membrane, nucleus and cytosol) extracted using GO terms listed in Supplementary Table 1.

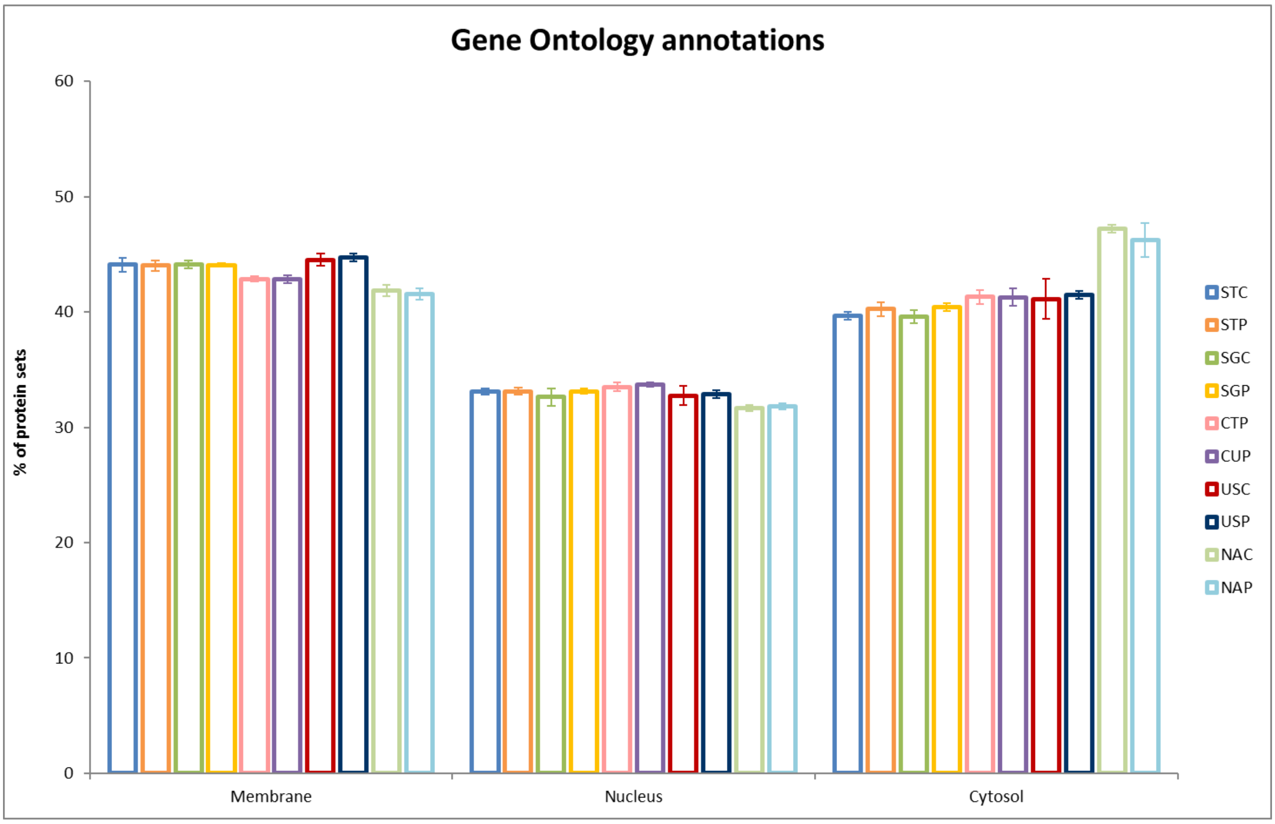

Supplementary Tables

Supplementary Table 1

Exhaustive list of GO terms considered for the distributions of the annotations membrane, nucleus and cytosol.

**"Membrane"**	external side of plasma membrane [GO:0009897]
	acrosomal membrane [GO:0002080]
	ER membrane protein complex [GO:0072546]
	membrane coat [GO:0030117]
	integral component of cytoplasmic side of endoplasmic reticulum membrane [GO:0071458]
	growth cone membrane [GO:0032584]
	mitochondrial outer membrane translocase complex [GO:0005742]
	lysosomal membrane [GO:0005765]
	sarcoplasmic reticulum membrane [GO:0033017]
	peroxisomal membrane [GO:0005778]
	mitochondrial intermembrane space protein transporter complex [GO:0042719]
	trans-Golgi network membrane [GO:0032588]
	intrinsic component of endoplasmic reticulum membrane [GO:0031227]
	endoplasmic reticulum membrane [GO:0005789]
	ciliary pocket membrane [GO:0020018]
	mitochondrial inner membrane [GO:0005743]
	cytoplasmic side of apical plasma membrane [GO:0098592]
	nuclear outer membrane [GO:0005640]
	intrinsic component of membrane [GO:0031224]
	mitochondrial intermembrane space [GO:0005758]
	nuclear inner membrane [GO:0005637]
	cytoplasmic side of early endosome membrane [GO:0098559]
	cell projection membrane [GO:0031253]
	early endosome membrane [GO:0031901]
	basolateral plasma membrane [GO:0016323]
	extrinsic component of endoplasmic reticulum membrane [GO:0042406]
	cytoplasmic side of membrane [GO:0098562]
	synaptic vesicle membrane [GO:0030672]
	platelet dense granule membrane [GO:0031088]
	extrinsic component of lysosome membrane [GO:0032419]
	autophagosome membrane [GO:0000421]
	clathrin-coated vesicle membrane [GO:0030665]
	microvillus membrane [GO:0031528]
	endoplasmic reticulum-Golgi intermediate compartment membrane [GO:0033116]
	extrinsic component of endosome membrane [GO:0031313]
	mitochondrial inner membrane presequence translocase complex [GO:0005744]
	plasma membrane bounded cell projection cytoplasm [GO:0032838]

	lumenal side of Golgi membrane [GO:0098547]<br>anchored component of synaptic vesicle membrane [GO:0098993]<br>membrane [GO:0016020]<br>cytoplasmic side of rough endoplasmic reticulum membrane [GO:0098556]<br>phagocytic vesicle membrane [GO:0030670]<br>perinuclear endoplasmic reticulum membrane [GO:1990578]<br>basement membrane [GO:0005604]<br>integral component of plasma membrane [GO:0005887]<br>stereocilium membrane [GO:0060171]<br>presynaptic active zone membrane [GO:0048787]<br>vesicle membrane [GO:0012506]<br>ruffle membrane [GO:0032587]<br>endosome to plasma membrane transport vesicle [GO:0070381]<br>extrinsic component of organelle membrane [GO:0031312]<br>extrinsic component of cytoplasmic side of plasma membrane [GO:0031234]<br>intracellular membrane-bounded organelle [GO:0043231]<br>endoplasmic reticulum tubular network membrane [GO:0098826]<br>ciliary membrane [GO:0060170]<br>intrinsic component of mitochondrial inner membrane [GO:0031304]<br>phagolysosome membrane [GO:0061474]<br>plasma membrane [GO:0005886]<br>Golgi membrane [GO:0000139]<br>azurophil granule membrane [GO:0035577]<br>presynaptic membrane [GO:0042734]<br>periciliary membrane compartment [GO:1990075]<br>anchored component of membrane [GO:0031225]<br>integral component of synaptic vesicle membrane [GO:0030285]<br>apical plasma membrane [GO:0016324]<br>integral component of mitochondrial outer membrane [GO:0031307]<br>synaptic membrane [GO:0097060]<br>integral component of autophagosome membrane [GO:0097637]<br>dendritic spine membrane [GO:0032591]<br>postsynaptic membrane [GO:0045211]<br>extrinsic component of Golgi membrane [GO:0090498]<br>extrinsic component of presynaptic active zone membrane [GO:0098891]<br>ER-mitochondrion membrane contact site [GO:0044233]<br>integral component of nuclear inner membrane [GO:0005639]<br>integral component of lumenal side of endoplasmic reticulum membrane [GO:0071556]<br>chromaffin granule membrane [GO:0042584]<br>cytoplasmic side of plasma membrane [GO:0009898]<br>membrane raft [GO:0045121]<br>platelet dense tubular network membrane [GO:0031095]<br>leading edge membrane [GO:0031256]

	sperm plasma membrane [GO:0097524]<br>zymogen granule membrane [GO:0042589]<br>integral component of endoplasmic reticulum membrane [GO:0030176]<br>integral component of mitochondrial membrane [GO:0032592]<br>brush border membrane [GO:0031526]<br>COPI-coated vesicle membrane [GO:0030663]<br>muscle cell projection membrane [GO:0036195]<br>melanosome membrane [GO:0033162]<br>mitochondrial membrane [GO:0031966]<br>invadopodium membrane [GO:0071438]<br>intrinsic component of external side of plasma membrane [GO:0031233]<br>extrinsic component of external side of plasma membrane [GO:0031232]<br>phagophore assembly site membrane [GO:0034045]<br>transport vesicle membrane [GO:0030658]<br>plasma membrane raft [GO:0044853]<br>filopodium membrane [GO:0031527]<br>neuronal cell body membrane [GO:0032809]<br>extrinsic component of mitochondrial outer membrane [GO:0031315]<br>vacuolar membrane [GO:0005774]<br>anchored component of external side of plasma membrane [GO:0031362]<br>Golgi cisterna membrane [GO:0032580]<br>endomembrane system [GO:0012505]<br>lamellipodium membrane [GO:0031258]<br>lateral plasma membrane [GO:0016328]<br>ER to Golgi transport vesicle membrane [GO:0012507]<br>integral component of Golgi membrane [GO:0030173]<br>organelle membrane [GO:0031090]<br>extrinsic component of membrane [GO:0019898]<br>membrane-bounded organelle [GO:0043227]<br>extrinsic component of mitochondrial inner membrane [GO:0031314]<br>nuclear membrane [GO:0031965]<br>integral component of peroxisomal membrane [GO:0005779]<br>mitochondrial outer membrane [GO:0005741]<br>secretory granule membrane [GO:0030667]<br>rough endoplasmic reticulum membrane [GO:0030867]<br>endosome membrane [GO:0010008]<br>organelle membrane contact site [GO:0044232]<br>apicolateral plasma membrane [GO:0016327]<br>dense core granule membrane [GO:0032127]<br>cytoplasmic vesicle membrane [GO:0030659]<br>recycling endosome membrane [GO:0055038]<br>symbiont-containing vacuole membrane [GO:0020005]<br>late endosome membrane [GO:0031902]

	basal plasma membrane [GO:0009925]<br>cytoplasmic side of endoplasmic reticulum membrane [GO:0098554]<br>extrinsic component of plasma membrane [GO:0019897]<br>mitochondrial inner membrane protein insertion complex [GO:0042721]<br>integral component of membrane [GO:0016021]<br>integral component of mitochondrial inner membrane [GO:0031305]<br>sperm head plasma membrane [GO:1990913]<br>Golgi-associated vesicle membrane [GO:0030660]<br>host cell presynaptic membrane [GO:0044231]<br>nuclear outer membrane-endoplasmic reticulum membrane network [GO:0042175]<br>nuclear pore transmembrane ring [GO:0070762]<br>dendrite membrane [GO:0032590]<br>intrinsic component of plasma membrane [GO:0031226]<br>photoreceptor outer segment membrane [GO:0042622]<br>postsynaptic endocytic zone membrane [GO:0098844]<br>virion membrane [GO:0055036]<br>presynaptic endocytic zone membrane [GO:0098835]<br>integral component of organelle membrane [GO:0031301]<br>intrinsic component of the cytoplasmic side of the plasma membrane [GO:0031235]<br>host cell mitochondrial intermembrane space [GO:0072492]<br>extrinsic component of presynaptic membrane [GO:0098888]<br>extrinsic component of postsynaptic membrane [GO:0098890]<br>AP-type membrane coat adaptor complex [GO:0030119]
**"Nucleus"**	nucleus<br>nucleus [GO:0005634]<br>female pronucleus [GO:0001939]<br>nucleus-vacuole junction [GO:0071561]<br>pronucleus [GO:0045120]<br>male germ cell nucleus [GO:0001673]<br>male pronucleus [GO:0001940]
**"Cytosol"**	cytosolic proteasome complex [GO:0031597]<br>cytosol<br>cytosol [GO:0005829]<br>cytosolic small ribosomal subunit [GO:0022627]<br>cytosolic large ribosomal subunit [GO:0022625]<br>cytosolic ribosome [GO:0022626]
